# Semantic annotation of biological concepts interplaying microbial cellular responses

**DOI:** 10.1186/1471-2105-12-460

**Published:** 2011-11-28

**Authors:** Rafael Carreira, Sónia Carneiro, Rui Pereira, Miguel Rocha, Isabel Rocha, Eugénio C Ferreira, Anália Lourenço

**Affiliations:** 1 IBB - Institute for Biotechnology and Bioengineering, Centre of Biological Engineering, University of Minho, Campus de Gualtar, 4710-057 Braga - PORTUGAL; 2 Department of Informatics/CCTC, University of Minho, Campus de Gualtar, 4710-057 Braga - PORTUGAL

## Abstract

**Background:**

Automated extraction systems have become a time saving necessity in Systems Biology. Considerable human effort is needed to model, analyse and simulate biological networks. Thus, one of the challenges posed to Biomedical Text Mining tools is that of learning to recognise a wide variety of biological concepts with different functional roles to assist in these processes.

**Results:**

Here, we present a novel corpus concerning the integrated cellular responses to nutrient starvation in the model-organism *Escherichia coli*. Our corpus is a unique resource in that it annotates biomedical concepts that play a functional role in expression, regulation and metabolism. Namely, it includes annotations for genetic information carriers (genes and DNA, RNA molecules), proteins (transcription factors, enzymes and transporters), small metabolites, physiological states and laboratory techniques. The corpus consists of 130 full-text papers with a total of 59043 annotations for 3649 different biomedical concepts; the two dominant classes are *genes *(highest number of unique concepts) and *compounds *(most frequently annotated concepts), whereas other important cellular concepts such as *proteins *account for no more than 10% of the annotated concepts.

**Conclusions:**

To the best of our knowledge, a corpus that details such a wide range of biological concepts has never been presented to the text mining community. The inter-annotator agreement statistics provide evidence of the importance of a consolidated background when dealing with such complex descriptions, the ambiguities naturally arising from the terminology and their impact for modelling purposes.

Availability is granted for the full-text corpora of 130 freely accessible documents, the annotation scheme and the annotation guidelines. Also, we include a corpus of 340 abstracts.

## Background

Due to its latest achievements, text mining, i.e. the automated extraction of information from electronically published sources, is receiving increasing interest from the scientific community [1-4]. Text mining has been accounted for in the literature curation pipelines of several databases devoted to cellular modelling, namely: Regulon's network of transcriptional regulation in *Escherichia coli *(*E. coli*) [5], the Open Regulatory Annotation database (ORegAnno) on *cis*-regulatory data [6], the KInetic Database (KID) [7] and the BRaunschweig ENzyme Database (BRENDA) [8] both covering kinetic enzyme information, the Mouse Genome Informatics (MGI) database [9], the STRING protein-protein interaction database [10], and a knowledge base on molecular mechanisms of bacterial enteropathogens [11]. Moreover, development efforts have been made to provide tools able to combine text mining techniques and manual curation into customised modelling workflows [4,12-14].

Here, we use our expertise on modelling microbial cellular processes to present a new set of annotated resources, addressing key modelling necessities, to be used by the text mining community. We provide corpora concerning the integrated cellular responses to nutrient starvation in the model-organism *E. coli*, in which a variety of metabolic and regulatory biological concepts with assorted functional roles is identified. Our annotation guidelines and our evaluation of Inter-Annotator Agreement (IAA) address biological issues such as: the specification of the biological concepts most relevant for studying cellular systems, how their basic functional roles can be fitted into a taxonomy of nominal classes and the terminological ambiguities that are likely to occur on microbial-related literature. Also, we analyse the impact of having annotators with different (levels of) expertise involved in such a process and describe the refinement of our annotation guidelines according to the outputs of annotator training.

The most relevant contents are available in supplementary material, namely: the corpus (available in multiple formats), data about the training stage (e.g. reports and guidelines), all IAA calculations and some other data considered useful to the community.

### Related work

Semantically annotated corpora are commonly used to train algorithms to extract information considered of interest. However, the construction of a corpus tends to be a laborious and time-consuming task that requires considerable domain expertise to guarantee the high-quality (correctness and meaningfulness) of the annotations. Evaluation efforts, such as BioCreative [15,16], TREC [17,18] and BioNLP [19], as well as individual text mining research projects, have tailored a number of corpora, freely available for the community.

Apart from their domain-specificities, corpora can be distinguished on the basis of the text segments included and the diversity of concepts involved. For a while, publishing policies and the costs associated with manual annotation dictated the use of abstracts or smaller segments (such as a randomised set of sentences) to construct corpora [20-25]. Although such segments hold a limited amount of information [26], this fact did not represent an actual research constraint as much had to be done in terms of training algorithms and building models. After all, state-of-the-art recognisers for key entities like genes, proteins and compounds are no more than five years old [27-31]. Meanwhile, the urge to develop fully equipped information extraction systems is demanding the construction and use of corpora of full-text documents [32-34], as well as the annotation of relationships/events [24,35-37].

Although the new corpora are of interest to Systems Biology applications and tools, most of them do not yet cover the desired holistic annotation of cellular processes. Except for a few recent works [24,35], existing corpora cover for a limited set of biological concepts (often, just *genes *and *proteins*) and annotation tends to focus on particular cellular processes (e.g. protein-protein interactions or transcriptional regulation), rather than to address integrated cellular processes (e.g. effects of transcriptional regulation over enzymatic reactions).

### Motivation and objectives

When aiding in the construction of cellular models, text mining systems have to deal with a wide variety of biological concepts with different functional roles. Information on gene expression and metabolic activities are at the core of cellular growth, development, reproduction and adaptability to environmental changes. Therefore, publications in the field often include the description of complex interactions involving genes, regulatory proteins (transcriptions factors and sigma factors), enzymes (and/or the catalytic reactions triggered by them), and metabolites (small molecules or compounds). Until now, a corpus that details a wide range of biological concepts has never been presented to the text mining community, thereby limiting the application of text mining on modelling tools. Particularly, one cannot forget that model reconstruction depends extensively on literature curation rather than the contents of databases [38] and thus, any means to enhance curation and integrate text mining facilities in modelling tools are of great interest to the community.

In this paper, we explore the issues that text mining systems will need to face in order to handle the knowledge contained within full-text articles. Since cellular modelling is one of our main research lines [39-43] and we have been evaluating text mining approaches in previous work [13,44], we are in a privileged position to provide both biologists' and modellers' perspectives about the construction of text mining resources. In particular, we investigate domain-specific semantic issues, namely ambiguities arising from terminology (e.g. polysemy and synonymy) and biological concepts playing multiple functional roles, by focussing on the manual identification of a set of key entities to cellular modelling within full-text articles. As final outcome, we provide two annotated corpora: a corpus of 130 full-text documents and a corpus of 340 abstracts.

In the remainder of this paper, we firstly introduce the key aspects of the annotation scheme, the annotation software used and the profiles and training of the annotators. Subsequently, we detail the construction of our corpus of full-text documents, quantifying and explaining annotation discrepancies and describing the post-processing that ensures the quality of the final corpus. Then, we perform a comparative analysis of our corpus of full-text documents and our corpus of abstracts regarding the number and diversity of biological concepts that have been annotated. Some conclusions and future work directions are stated at the end.

## Methods

This section characterises our corpora and describes the preparatory work required prior to their annotation. Since the preparatory work, annotation and analysis of our corpus of full-texts and our corpus of abstracts have been performed in a similar way, we describe the process applied to the corpus of full-texts, while the data corresponding to the corpus of abstracts is provided in supplementary material.

### Corpora characterisation

The candidate documents for our work were retrieved from PubMed, using the keywords ("Escherichia coli" and "stringent response"), in January 2010. These candidate documents were screened for relevance by two of the authors with biological expertise, resulting in a set of 340 documents. From this set, we constructed a corpus of 340 abstracts and a corpus of 130 open-access full-texts.

The process of annotation of the two corpora was the same. Specifically, annotators followed the same annotation scheme and guidelines, the quality of their annotations was quantified similarly and identical post-processing ensured the high-quality of the final annotations. The effort expended by the two annotators amounted to a total of approximately 1188 person hours (equivalent to 6.75 person months).

The corpora are delivered in two XML-based formats: inline annotation and stand-off annotation. Individual annotations comprise the text span and its offsets (referring to the full extent of the text), a category from our hierarchy of biological concept types (see Annotation scheme section), and, whenever possible, the database identifier (i.e. an identifier from EcoCyc [45] or PSI-MI [46]) and the associated common name. In supplementary material we provide the list of PubMed identifiers (PMIDs) of the documents that compose our corpora (additional file "List of Documents") as well as the annotated corpus of full-text documents (additional files "FulltextsCorpus_Inline" and "FulltextsCorpus_Standoff") and the annotated corpus of abstracts (additional files "AbstractsCorpus_Inline" and "AbstractsCorpus_Standoff").

### Annotation scheme

**Figure 1:**
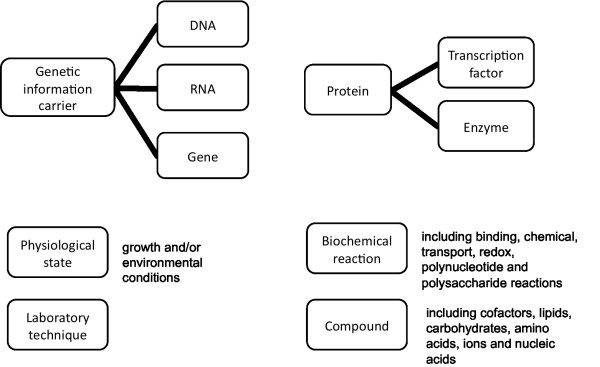
**The hierarchical structure of the biological concepts**. The hierarchy covers biological concepts that characterize the organizational structure of microbial systems, the physiological conditions that affect them and the laboratory techniques used to identify their underlying properties. **Note: **The supercategory *genetic information carrier *is used only for organisational purposes, i.e. the category is never used as an annotation category.

We have arranged biological concepts into the following semantic categories (Figure 1): *genetic information carrier *(which includes the categories *gene*, *dna *and *rna*), *protein *(which includes the categories *enzyme *and *transcription factor*), *compound*, *biochemical reaction*, *physiological state *and *laboratory technique*. Besides common biological concepts, we are interested in growth and environmental conditions or changes, i.e. conditions that somehow trigger regulatory actions at either the transcriptional or the metabolic level (e.g. cell adaptation to osmotic variations or nutrient deprivation). We are also interested in the laboratory techniques used to identify biological entities (e.g. mass spectrometry) and characterise the underlying properties of the biological systems involving these entities (e.g. *in vitro *enzymatic assays).

Each biological concept has a number of possible names associated (i.e. all the text forms that annotators have recognised in the documents), but has unambiguous meaning and can be associated with a unique database identifier. Namely, we have used the EcoCyc knowledge base, a key resource for *E. coli *studies, to organise molecular information and the Molecular Interactions (PSI-MI) ontology to index laboratory techniques.

To attain high quality annotations, we have produced a detailed set of annotation guidelines that describe the biological concepts pertaining to each semantic category, and provide clear positive and negative examples of concept annotation. The structure and content of these guidelines were iteratively refined in discussion with domain experts and annotators, via group discussions after the training cycles and after the post-processing of the corpora. The final guidelines of annotation can be found in additional file "Final Guidelines" in supplementary material.

### Annotation software

The retrieval of documents and the subsequent manual annotation was performed using @Note, a workbench for Biomedical Text Mining [47]. This workbench enables keyword-based PubMed querying and document retrieval. Moreover, it provides a user-friendly interface for document visualisation and annotation: a colour-based scheme keeps visual track of any annotation editing, whilst another panel shows updated statistics on document and corpus annotation; a browsing mechanism allows the annotator to query well-known databases about name variants; and, a basic statistics feature characterises the ongoing annotation in terms of the number of concepts and associated annotations for each category. See additional file "Software Snapshots" in supplementary material for illustrative snapshots of the process.

### Annotator profiling

The entanglement of biological concepts in the description of cellular processes is not straightforward to follow by those who do not have enough familiarity with biochemical pathways and regulatory cascades. Therefore, although the availability of annotators is often quite limited, it is important to establish the minimum level of expertise to be required for the production of such a corpus and the level of detail that annotation guidelines have to encompass in order to ensure the quality of annotation.

To perform this exercise, and given the availability of human resources, we chose two annotators with different levels of expertise. A student finishing a PhD thesis on the stringent response of *E. coli *(designated as annotator **A**) and a student who has recently started his PhD in *E. coli *bioprocesses (designated as annotator **B**). Both annotators present near-native competence in English and have solid background in Biology, but one differs from the other in terms of familiarity with microbial cellular processes and the *E. coli *stress phenomena.

### Annotator training and quality control

Our training process included a preliminary session and three training cycles. First, we introduced the annotation tool and the task, exemplifying the annotation guidelines with some positive and negative examples of the biological concepts to be annotated (see additional file "Initial Guidelines" in supplementary material). Next, we selected a subset of 45 documents to be presented to both annotators and initiated the training cycles. Each training cycle included the annotation of the corpus based on the last updates on the guidelines (see additional files "Report after cycle 1" and "Report after cycle 2" in supplementary material), the calculation of the rates of agreement between annotators, the examination of the discrepancies and the refinement of the guidelines.

To quantify the improvement in the rates of agreement, we calculated the F-score, a common metric in IAA evaluations [24,28,35].

(1) $$F-score=2\times \frac{precison\times recall}{precision+recall}$$

(2) $$Precision=\frac{number\;of\;identical\;entities\;in\;set\;A\;and\;set\;B}{number\;of\;entities\;in\;set\;A}$$

(3) $$Recall\;=\frac{number\;of\;identical\;entities\;in\;set\;A\;and\;set\;B}{number\;of\;entities\;in\;set\;B}$$

**Legend**: Set A refers to the set of annotations produced by annotator A and set B refers to the set of annotations produced by annotator B. Also, note that recall(set A, set B) = precision(set B, set A) [48].

Then, two of the authors (with extensive biological expertise in the field) examined the annotation discrepancies and, prior to the next cycle of annotation, the group discussed the observed ambiguities/glitches and revised the annotation guidelines accordingly (e.g. by introducing more positive and negative examples of the different concepts).

In general, the trend was for the rates of agreement to improve between training cycles (Table 1). At the end of the last cycle, most agreement levels were approximate to or above 50%, which we consider to be acceptable regarding the different levels of expertise of our annotators and the expected complexity in assigning some of the concepts.

**Table 1:** Inter-annotator agreement during training

	Training cycle 1	Training cycle 2	Training cycle 3
*dna*	-	5.74%	21.74%

*rna*	-	55.81%	65.63%

*gene*	69.39%	63.26%	83.08%

*protein*	-	52.71%	48.45%

*enzyme*	41.03%	53.78%	65.28%

*transcription factor*	0%	38.46%	20.51%

*compound*	45.28%	65.36%	71.54%

*biochemical reaction*	0%	0%	0%

*physiological state*	27.85%	40.94%	42.51%

*laboratory technique*	23.01%	48.98%	48.52%

Columns report the F-scores obtained for each semantic category after a cycle of training. Note that the annotation of concepts for categories *dna *and *rna *(under the supercategory *genetic information carrier*, which also includes the category *gene*) and *protein *(that aggregates the subcategories *enzyme *and *transcription factor*) was considered only after the first training cycle.

Indeed, we concluded that the slight decrease of the rates of agreement for *protein *and *transcription factor *in the last cycle is caused by different levels of experience on gene regulation and also, the distinction between *biochemical reaction *and *enzyme *is unclear to less experienced annotators.

## Results and discussion

After the training period, the corpus of 130 full-texts and the corpus of 340 abstracts were constructed (all documents have been annotated by both annotators following the same collection of rules detailed in additional file "Final Guidelines" in supplementary material).

In this section, we detail the process of annotation for the corpus of 130 full-texts, examining the IAA scores obtained and explaining the post-processing that was executed to reach a biologically consistent consensus and eliminate annotation inconsistencies. Further on, we provide the general statistics of the final corpora and perform a comparison regarding the distribution of concepts per semantic category.

### Quality control

Since, during the training period, we concluded that part of the annotation inconsistencies were due to the different levels of expertise of the annotators, we were expecting similar problems at the annotation of the final corpus (Table 2). Indeed, although the rates of agreement for most categories are fairly good (approximately or above 50%), the only category that achieves high agreement is *gene*, while categories such as *transcription factor *and *dna *(about concepts related to regulatory functions) present lower scores.

**Table 2:** Inter-annotator agreement for the 130 full-texts

	Final F-score
*dna*	13.22%

*rna*	59.69%

*gene*	91.78%

*protein*	42.15%

*enzyme*	63.33%

*transcription factor*	28.13%

*compound*	63.90%

*biochemical reaction*	0%

*physiological state*	46.50%

*laboratory technique*	38.34%

F-scores were estimated for each semantic category.

Thus, we decided to explore the nature of the discrepancies by detailing the assignments of the annotators per semantic category (Table 3). Specifically, we had a round of group meetings where we examined discrepancies in category assignment, assignments to fine-grained categories and differences in the number of annotations.

**Table 3:** Annotator assignments per category

	Annotator B											
		
		*dna*	*rna*	*gene*	*protein*	*enzyme*	*transcription factor*	*compound*	*biochemical reaction*	*laboratory technique*	*physiological state*	None
**Annotator A**	***dna***	**8** **(2316)**	0	0	0	0	0	0	0	0	0	58 (204)

	***rna***	0	57 **(9078)**	0	0	0	0	0	0	0	1 (64)	23 (432)

	***gene***	0	0	**1066** **(16250)**	3 (68)	0	0	3 (534)	0	0	0	59 (276)

	***protein***	0	0	4 (564)	**55** **(3396)**	5 (18)	0	2 (64)	0	0	0	53 (261)

	***enzyme***	0	0	0	3 (12)	**196** **(5457)**	0	0	0	0	0	52 (219)

	***transcription factor***	0	1 (20)	2 (6)	7 (1510)	0	**9** **(231)**	0	0	0	0	27 (431)

	***compound***	3 (170)	0	1 (8)	0	1 (68)	0	**369** **(36857)**	0	0	0	75 (683)

	***biochemical reaction***	0	0	0	0	48 (1425)	0	0	**0**	0	0	13 (48)

	***laboratory technique***	0	0	0	1 (18)	1 (6)	0	2 (183)	0	**136** **(3495)**	1 (40)	121 (852)

	***physiological state***	0	0	0	0	0	0	0	0	0	**97** **(8361)**	210 (5397)

	**None**	44 (1555)	52 (130)	119 (529)	73 (440)	117 (941)	9 (30)	330 (2163)	1 (1)	187 (575)	100 (786)	

Cells represent the assignments of both annotators in terms of the number of biological concepts and the corresponding number of annotations (depicted between parentheses). Consensual assignments, i.e. assignments to the same category, are depicted at the diagonal of the table in bold; discrepancies in category assignment are indicated by non-diagonal cells; and the pseudo-category "None" represents all assignments made by only one of the annotators. For instance, the top left hand cell indicates that annotators agreed on 8 biological concepts for the category *dna*, corresponding to a total of 2316 annotations.

#### Polysemy

Situations of polysemy (i.e. when a name, one or more words, may be used to denote different biological concepts belonging to different semantic categories) caused many category disagreements. Namely, we observe this problem between the categories *gene *and *compound*, *gene *and *protein*, *protein *and *compound*, and *enzyme *and *biochemical reaction*.

For example: names such as "leu", "mal" and "fum" are names of genes but are also acronyms of the compounds "leucine", "maltose" and "fumarate"; in the domain of our corpus, the name "stringent factor" is a synonym for both the compound "ppGpp" and the enzyme "RelA"; the name "gpp" is a synonym for a gene coding for the enzymes "guanosine-5'-triphosphate, 3'-diphosphate pyrophosphatase" and "xanthine-guanine phosphoribosyltransferase" and the compound "geranyl diphosphate"; and the name "glyD" is a synonym for a geneand an enzyme. Also, the names of reactions are easily misinterpreted with the names of enzymes, because the latest are mainly derived from the designation of the respective catalysing biochemical reaction ending in 'ase' and its substrate name (e.g. sulfate adenylyltransferase catalyses the transfer of phosphorus-containing nucleotide groups to sulphate (substrate), i.e. performs the adenylyltransferase activity).

#### Synonyms/name variants

The high degree of synonymy found in biological vocabularies represents a challenge to the comprehensiveness of the annotation. Indeed, the use of different names to denote the same biological concept requires a certain familiarity with the field, as many synonyms are not catalogued in databases and many name variants are only associated with a biological concept in a very specific context. Just to exemplify, during the annotation of our corpus, we have found 27 name variants of the compound "ppGpp", 23 name variants of the enzyme "RelA", and 5 name variants for the transcription factor "CRP-cAMP".

#### Fine-grained semantics

Annotator **B **assigned many *transcription factor *and *enzyme *concepts to *protein*. For example, non-obvious enzyme names (e.g. "penicillin binding protein 2", and the synonyms "OmpT" and "outer membrane protein 3b") and transcription factors (e.g. "ntrc" that designates the NtrC transcriptional dual regulator and "ole" that designates the FadR DNA-binding transcriptional dual regulator) were annotated as *protein*.

#### Misclassification of biological concepts

Some category disagreements were seen to be due to misclassifications by one of the annotators. The reasons behind these misclassifications are somewhat difficult to unravel, but it could be attributed to misinterpretations in specific biological contexts. For example, we found out that annotator **B **misclassified some compounds as *dna *(e.g. "mal" that is an abbreviation of "maltose", a compound that is used as a carbon source by many organisms) or as *enzyme *(e.g. "luciferin" which is a class of small-molecules that are oxidized in the presence of the enzyme luciferase to produce oxyluciferin and energy in the form of light). Annotator **B **also annotated most transcription units (e.g. "thrAC", "tauABCD", and "sucACD") and operons (e.g. "gltB operon" and " ftsQAZ operon") as *gene *and misclassified some laboratory techniques(e.g. "sonication" as *physiological state*, "autoradiography" as *compound *and "dideoxysequencing reaction" as *biochemical reaction*).

#### Exclusively annotated concepts

The extent of biological concepts that were exclusively annotated by each of the annotators is large and fall particularly into categories like *compound*, *laboratory technique *and *physiological state*. Annotator **B **tended to annotate more exclusive terms than annotator **A**, in particular in *gene *category (119 and 59 exclusive terms, respectively), *enzyme *category (117 and 52 exclusive terms, respectively) and *compound *category (330 and 75 exclusive terms, respectively). It was observed that Annotator **B **did not strictly follow some of the guidelines and annotated genes that do not belong to *E. coli *or were introduced via genetic transformations (e.g. the gene *bmp*A is from the bacteria *Borrelia burgdorferi*), or terms that should be assigned to the *biochemical reaction *in the *enzyme *category (e.g. hydrolase is not an enzyme but the activity performed by several enzymes) and also, compounds that do not participate in biochemical reactions within *E. coli *cells, but are used in laboratory assays (e.g. azthreonam that is a synthetic monocyclic beta-lactam antimicrobial agent).

In categories like *laboratory technique *and *physiological state*, the number of concepts exclusively annotated was roughly even for both annotators. Apparently, the annotation of these biological concepts is highly dependent on the background knowledge of the annotators. As observed, annotator **A **annotated many more concepts in the *physiological state *category. This can be explained by the fact that he is more familiar with the case study (i.e. the stringent response in *E. coli*) compared to annotator **B**. Likewise, the extent of exclusive terms assigned to the *laboratory technique *category was conditioned by the experience of each annotator. While annotator **B **has considered more laboratory techniques related to biochemistry and genetics (e.g. reverse transcriptase mapping or ribonuclease protection assays), annotator **A **annotated more techniques that are related to preparative or analytical techniques for measuring analytes (e.g. chip immunoprecipitation or capillary electrophoresis).

### Post-processing

After identifying the main issues affecting annotation consistency, we undertook a final round of group sessions to resolve them. Most inconsistencies were resolved in favour of the more experienced annotator, specifically inconsistencies related to the above mentioned fine-grained category assignments, exclusively annotated concepts, and polysemy and synonymy situations.

To guarantee the high-quality of the final corpus we have revised the annotations as follows:

*• biochemical reaction*: since the disagreements throughout the whole process of training and corpus annotation were considerable, it was decided to not include this biological concept in the final corpus. Therefore, in addition to the previous rules regarding the transition of terms between the *enzyme *and *biochemical reaction *concepts, it was determined to eliminate this biological concept from the final corpus.

*• compound*: the level of exclusive terms included in the 130 full-text corpus by the Annotator **B **is considerably higher when compared to those annotated by Annotator **A**. The annotation of compounds that are uniquely used in biochemical assays (e.g. EDTA or acetonitrile) was previously decided (see additional file "Report after cycle 1") to be disregarded. However, some of these terms were still assigned as compounds and, consequently, were filtered out;

*• gene*: similarly to what was seen in compound annotations, the addition of genes that are coming from other biological sources other than *E. coli *must be disregarded. Thus, it was agreed that any term that refers to a gene from another organism will be omitted (e.g. bmpA, bmpB or bmpD designate genes from *Borrelia burgdorferi *and should not be annotated).

*• laboratory technique*: the number of exclusive terms annotated by both annotators exceeds the number of concepts in which annotators agreed. This denotes the importance of the annotator expertise at specific research areas as explained before. In this case, it was decided to join annotations from both annotators;

*• physiological state*: annotations on this category were also disparate when analysing exclusive terms from both annotators and were most likely a consequence of the level of expertise of the two annotators. Since annotator **A **was more familiar with the case study (i.e. the stringent response in *E. coli*) many more terms were classified. Nevertheless, annotator **B **assigned some terms that were missed by annotator **A **and it was decided to include them in the final corpus.

### Final corpus statistics

After ensuring the consistency of the annotations, our corpus of 130 full-text documents comprises 59043 annotations, corresponding to 3649 unique biological concepts, distributed according to the categories of our scheme as shown in Table 4.

**Table 4:** General statistics about the corpus of full-text documents

Categories		#concepts	# annotations	% concepts	% annotations	Annotation Frequency	Concept Distribution
Genetic Information Carrier	*dna*	126	3771	3.45%	6.39%	29.93	8.87
	
	*rna*	119	3970	3.26%	6.72%	**33.36**	8.38
	
	*gene*	**1175**	8770	32.20%	14.85%	7.46	**82.75**

Protein	*protein*	175	2332	4.80%	3.95%	13.33	28.69
	
	*enzyme*	388	4025	10.63%	6.82%	10.37	63.61
	
	*transcription factor*	47	1434	1.29%	2.43%	30.51	7.70

*compound*		767	**21414**	21.02%	36.27%	27.92	

*physiological state*		403	10166	11.04%	17.22%	25.23	

*laboratory technique*		449	3161	12.30%	5.35%	7.04	

**Total**		**3649**	**59043**	**100%**	**100%**		

The first statistics depict the number and percentage of biological concepts and associated annotations, and the frequency of annotations per category. Besides individual categories, there are hierarchically structured annotation categories: the categories *dna*, *rna *and *gene *belong to the supercategory *genetic information carrier*; and the categories *protein*, *enzyme *and *transcription fac*tor are subcategories of *protein*. For these categories, the concept distribution of a category is then calculated by dividing the number of biological concepts assigned to the category per the total number of biological concepts assigned to its supercategory.

**Legend: **The symbol "#" stands for "number of" and the symbol "%" stands for "percentage of". Frequencies are calculated as follows:

$$concept\;distribution=\frac{number\;of\;biological\;concepts\;in\;category}{total\;number\;of\;biological\;concepts\;in\;supercategory}$$

$$annotation\;frequency=\frac{number\;of\;annotations\;in\;category}{number\;of\;biological\;concepts\;in\;category}$$

*Gene *and *compound *are the categories with the largest number of biological concepts annotated. This is explained by the fact that most activities related to metabolism and gene expression are described to some extent by concepts of these two categories. Regarding the ratio of biological concepts, the category *compound *is about 10% below the category *gene*, but its ratio of annotations is almost 20% higher than the category *gene*. This means that we have annotated more *gene *concepts but comparatively, the documents contain more mentions of *compound *concepts. Once again, this is explained by the fact that *E. coli *stringent response is triggered by compounds, (p)ppGpp, and then influences the expression of many genes. In addition, the low ratios of the categories *protein, enzyme *and *transcriptional factor *(below 5%, 11% and 2%, respectively), and subcategories *rna *and *dna *in the *genetic information carrier *supercategory (around 3%) confirm that most of the discussion on cellular responses is centred on *genes *and *compounds*.

It is also important to notice that the category *physiological state *represents 11% of the biological concepts in the corpus and 17% of the annotations in the corpus, with a high frequency of annotation per concept (around 25 annotations). Additionally, in the *laboratory technique *category, encompassing biological concepts that establish a mechanistic link between genes, compounds and proteins, the ratio of biological concepts is about 12% but the ratio of annotations is less than half of this, with a frequency of 7 annotations per technique.

### Comparative analysis of full-text and abstract assignments

Compared to full-texts, abstracts are less rich and complex text segments. Abstracts tend to mention only key biological concepts and the text is quite concise. So, we would expect that abstract annotation would be significantly less affected by differing levels of expertise.

To be able to draw a fair comparison, we have used only the 130 documents that are common to both corpora. We consider the improvement of the rates of agreement during the training period (which only covered 45 of the 130 documents) and the rate of agreement for the 130 documents, and the final distribution of biological concepts per semantic category (Table 5). During training, abstracts have a greater improvement of the rates for most categories, except for the subcategories of *gene*, i.e. *dna *and *rna*, and the categories *compound *and *physiological state*. This could be explained by the same problems found during training on full papers, where the assignment of transcription units or operons as *gene *is confused with *dna *(e.g. operon cyoABCDE), and the inclusion of compounds that are not participants in the metabolism, but are chemical compounds used in assays (e.g. EDTA, a chelating agent used for gel electrophoresis), were detected. These misclassifications would decrease the IAA measurements.

**Table 5:** General statistics about agreement rates and concept assignments for the two corpora

	Abstracts		Full-texts	
	
	F-scores	Final number of biological concepts	F-scores	Final number of biological concepts
*dna*	30.77%	25	13.22%	126

*rna*	81.82%	32	59.69%	119

*gene*	87.84%	73	91.78%	1175

*protein*	45.16%	35	42.15%	175

*enzyme*	70.18%	67	63.33%	388

*transcription factor*	20%	17	28.13%	47

*compound*	83.09%	188	63.90%	767

*biochemical reaction*	0%	-(*)	0%	-(*)

*physiological state*	46.63%	145	46.50%	403

*laboratory technique*	75.27%	58	38.34%	449

The F-score columns refer to the F-score values achieved for the 130 documents after training and before post-processing; and the final number of biological concepts is calculated after post-processing.

(*) This biological concept was not included in the final corpora. See the Post-processing sub-section for more details.

When constructing the corpora of 130 documents, the rates of agreement achieved for abstracts are significantly higher than those achieved for full-texts, except for *gene *(4% less) and *transcription factor *(8% less) assignments. For these biological concepts, one of the annotators assigned fewer terms, resulting in lower agreement rates. It was found that most terms were annotated as the supercategory (i.e., *protein*) by one of the annotators, while the other discriminated the functional role of those proteins (e.g. CRP and Lrp that DNA-binding transcriptional regulators). The same happened when annotating full-texts, but the proportion of these misclassified terms was lower, which contributed to a slightly increased F-score.

## Conclusions

We have designed a schema and a set of guidelines in support of the semantic annotation of microbial cellular responses. We have produced a corpus of 130 free-access full-texts with a total of 59043 annotations, corresponding to 3649 unique biomedical concepts.

Through discrepancy analysis of the corpus, we have pin-pointed the most problematic issues for annotators, both in terms of terminological and background-related issues. Our results show that high levels of agreement (over 90%) can only be achieved for the *gene *category. The average agreement rate for most of the other categories is around 50%. The exceptions are the categories *dna *and *biochemical reaction *that have very low levels of agreement. So, it is interesting to notice that even amongst experienced researchers, annotation is still subjective and highly dependent on whether or not the researcher is familiar with the cellular processes under annotation. Specifically, such high familiarity is required in the annotation of different types of biological concepts (often sharing common names) that play distinct cellular roles depending on the context of the statement. This is considered a major concern for the purpose of reconstructing cellular models. The size of the model and its complexity may hide some mis-annotations (or mis-extractions) and lead to false biological interpretations.

This full-text corpus is suitable for use in the validation of the ability of information extraction tools in ambiguous contexts. Moreover, since the corpus encompasses a wide variety of biological concepts at the core of cellular responses, we believe that it may be a useful resource in the development of text mining tools supporting the reconstruction of cellular models. The corpus is freely accessible at http://sysbio.uminho.pt/corpus_ecoli.

## Authors' contributions

All authors participated in the preparation of the manuscript. AL supervised all steps of the work. SC and RP performed the annotations, whilst ECF and IR prepared the annotation scheme and oriented training discussion, covering for key biological issues. RC and MR provided technical support during the training phase and were responsible for compiling corpus statistics and evaluating IAA. The analysis of annotation discrepancies derived from a joint effort. All authors read and approved the final manuscript.

## References

[B1] Harmston N Filsell W Stumpf MP What the papers say: Text mining for genomics and systems biology Hum Genomics 2010 5 17 29 21106487 10.1186/1479-7364-5-1-17 PMC3500154

[B2] Krallinger M Leitner F Valencia A Analysis of biological processes and diseases using text mining approaches Methods Mol Biol 2010 593 341 382 10.1007/978-1-60327-194-3_16 19957157

[B3] Kowald A Schmeier S Text mining for systems modeling Methods Mol Biol 2011 696 305 318 10.1007/978-1-60761-987-1_19 21063956

[B4] Kemper B Matsuzaki T Matsuoka Y Tsuruoka Y Kitano H Ananiadou S Tsujii J PathText: a text mining integrator for biological pathway visualizations Bioinformatics 2010 26 i374 i381 10.1093/bioinformatics/btq221 20529930 PMC2881405

[B5] Rodriguez-Penagos C Salgado H Martinez-Flores I Collado-Vides J Automatic reconstruction of a bacterial regulatory network using Natural Language Processing BMC Bioinformatics 2007 8 10.1186/1471-2105-8-293 PMC1964768 17683642

[B6] Griffith OL Montgomery SB Bernier B Chu B Kasaian K Aerts S Mahony S Sleumer MC Bilenky M Haeussler M Griffith M Gallo SM Giardine B Hooghe B Van LP Blanco E Ticoll A Lithwick S Portales-Casamar E Donaldson IJ Robertson G Wadelius C De BP Vlieghe D Halfon MS Wasserman W Hardison R Bergman CM Jones SJ ORegAnno: an open-access community-driven resource for regulatory annotation Nucleic Acids Res 2008 36 D107 D113 10.1093/nar/gkn457 18006570 PMC2239002

[B7] Heinen S Thielen B Schomburg D KID--an algorithm for fast and efficient text mining used to automatically generate a database containing kinetic information of enzymes BMC Bioinformatics 2010 11 375 10.1186/1471-2105-11-375 20626859 PMC2912889

[B8] Scheer M Grote A Chang A Schomburg I Munaretto C Rother M Sohngen C Stelzer M Thiele J Schomburg D BRENDA, the enzyme information system in 2011 Nucleic Acids Res 2011 39 D670 D676 10.1093/nar/gkq1089 21062828 PMC3013686

[B9] Bult CJ Kadin JA Richardson JE Blake JA Eppig JT The Mouse Genome Database: enhancements and updates Nucleic Acids Res 2010 38 D586 D592 10.1093/nar/gkp880 19864252 PMC2808942

[B10] Jensen LJ Kuhn M Stark M Chaffron S Creevey C Muller J Doerks T Julien P Roth A Simonovic M Bork P von MC STRING 8--a global view on proteins and their functional interactions in 630 organisms Nucleic Acids Res 2009 37 D412 D416 10.1093/nar/gkn760 18940858 PMC2686466

[B11] Zaremba S Ramos-Santacruz M Hampton T Shetty P Fedorko J Whitmore J Greene JM Perna NT Glasner JD Plunkett G Shaker M Pot D Text-mining of PubMed abstracts by natural language processing to create a public knowledge base on molecular mechanisms of bacterial enteropathogens BMC Bioinformatics 2009 10 177 10.1186/1471-2105-10-177 19515247 PMC2704210

[B12] Spasic I Simeonidis E Messiha HL Paton NW Kell DB KiPar, a tool for systematic information retrieval regarding parameters for kinetic modelling of yeast metabolic pathways Bioinformatics 2009 25 1404 1411 10.1093/bioinformatics/btp175 19336445

[B13] Lourenço A Carreira R Carneiro S Maia P Glez-Peña D Fdez-Riverola F Ferreira EC Rocha I Rocha M @Note: A workbench for Biomedical Text Mining Journal of Biomedical Informatics 2009 10.1016/j.jbi.2009.04.002 19393341

[B14] Kano Y Dobson P Nakanishi M Tsujii J Ananiadou S Text mining meets workflow: linking U-Compare with Taverna Bioinformatics 2010 26 2486 2487 10.1093/bioinformatics/btq464 20709690 PMC2944208

[B15] Krallinger M Valencia A BioCreative III, PPI Task 2010 http://www.biocreative.org/tasks/biocreative-iii/ppi/

[B16] Leitner F Mardis SA Krallinger M Cesareni G Hirschman LA Valencia A An Overview of BioCreative II.5 IEEE/ACM Trans Comput Biol Bioinform 2010 7 385 399 20704011 10.1109/tcbb.2010.61

[B17] Hersh W Bhupatiraju RT TREC Genomics Track Overview 2003 14 23

[B18] Hersh W Bhupatiraju RT Ross L Johnson P Cohen AM Kraemer DF TREC 2004 Genomics Track Overview 2004 13 31 10.1186/1747-5333-1-3 PMC1440302 16722581

[B19] Kim JD Ohta T Pyysalo S Kano Y Tsujii J Overview of BioNLP'09 shared task on event extraction 2009 1 9

[B20] Kim JD Ohta T Tateisi Y Tsujii J GENIA corpus--semantically annotated corpus for bio-textmining Bioinformatics 2003 19 Suppl 1 i180 i182 10.1093/bioinformatics/btg1023 12855455

[B21] Pyysalo S Ginter F Heimonen J Bjorne J Boberg J Jarvinen J Salakoski T BioInfer: a corpus for information extraction in the biomedical domain BMC Bioinformatics 2007 8 10.1186/1471-2105-8-50 PMC1808065 17291334

[B22] Pyysalo S Airola A Heimonen J Bjorne J Ginter F Salakoski T Comparative analysis of five protein-protein interaction corpora BMC Bioinformatics 2008 9 10.1186/1471-2105-9-S3-S6 PMC2349296 18426551

[B23] Tanabe L Xie N Thom LH Matten W Wilbur WJ GENETAG: a tagged corpus for gene/protein named entity recognition BMC Bioinformatics 2005 6 10.1186/1471-2105-6-S1-S3 PMC1869017 15960837

[B24] Thompson P Iqbal SA McNaught J Ananiadou S Construction of an annotated corpus to support biomedical information extraction BMC Bioinformatics 2009 10 10.1186/1471-2105-10-349 PMC2774701 19852798

[B25] Leitner F Krallinger M Cesareni G Valencia A The FEBS Letters SDA corpus: a collection of protein interaction articles with high quality annotations for the BioCreative II.5 online challenge and the text mining community FEBS Lett 2010 584 4129 4130 10.1016/j.febslet.2010.08.026 20728446

[B26] Cohen KB Johnson HL Verspoor K Roeder C Hunter LE The structural and content aspects of abstracts versus bodies of full text journal articles are different BMC Bioinformatics 2010 11 492 10.1186/1471-2105-11-492 20920264 PMC3098079

[B27] Settles B ABNER: an open source tool for automatically tagging genes, proteins and other entity names in text Bioinformatics 2005 21 3191 3192 10.1093/bioinformatics/bti475 15860559

[B28] Corbett P Batchelor C Teufel S Annotation of chemical named entities BioNLP 2007: Biological, translational, and clinical language processing 2007 57 64

[B29] Mika S Rost B NLProt: extracting protein names and sequences from papers Nucleic Acids Res 2004 32 W634 W637 10.1093/nar/gkh427 15215466 PMC441565

[B30] Tsuruoka Y Tateishi Y Kim JD Ohta T McNaught J Ananiadou S Tsujii J Developing a robust part-of-speech tagger for biomedical text Advances in Informatics, Proceedings 2005 3746 382 392 10.1007/11573036_36

[B31] Leaman R Gonzalez G BANNER: an executable survey of advances in biomedical named entity recognition Pac Symp Biocomput 2008 652 663 18229723

[B32] McIntosh T Curran JR Challenges for automatically extracting molecular interactions from full-text articles BMC Bioinformatics 2009 10 311 10.1186/1471-2105-10-311 19778419 PMC2761905

[B33] Wang H Huang M Zhu X Extract interaction detection methods from the biological literature BMC Bioinformatics 2009 10 Suppl 1 S55 10.1186/1471-2105-10-S1-S55 19208158 PMC2648772

[B34] Gerner M Nenadic G Bergman CM LINNAEUS: a species name identification system for biomedical literature BMC Bioinformatics 2010 11 85 10.1186/1471-2105-11-85 20149233 PMC2836304

[B35] Kim JD Ohta T Tsujii J Corpus annotation for mining biomedical events from literature BMC Bioinformatics 2008 9 10.1186/1471-2105-9-10 PMC2267702 18182099

[B36] Rajagopala SV Goll J Gowda ND Sunil KC Titz B Mukherjee A Mary SS Raviswaran N Poojari CS Ramachandra S Shtivelband S Blazie SM Hofmann J Uetz P MPI-LIT: a literature-curated dataset of microbial binary protein--protein interactions Bioinformatics 2008 24 2622 2627 10.1093/bioinformatics/btn481 18786976 PMC2720748

[B37] Oda K Kim JD Ohta T Okanohara D Matsuzaki T Tateisi Y Tsujii J New challenges for text mining: mapping between text and manually curated pathways BMC Bioinformatics 2008 9 10.1186/1471-2105-9-S3-S5 PMC2352872 18426550

[B38] Rocha I Forster J Nielsen J Osterman AL, Gerdes S Design and application of genome-scale reconstructed metabolic models Microbial Gene Essentiality: Protocols and Bioinformatics 2008 416 409 431 *In series: Methods in Molecular Biology* 10.1007/978-1-59745-321-9_29 18392985

[B39] Pinto JP Dias O Lourenço A Carneiro S Ferreira EC Rocha I Rocha M Data Integration Issues in the Reconstruction of the Genome-Scale Metabolic Model of *Zymomonas Mobillis* Advances in Soft Computing 2009 92 101

[B40] Mendes R Lourenço A Carneiro S Ferreira EC Rocha I Rocha M A Framework for the Integrated Analysis of Metabolic and Regulatory Networks The 8th IEEE International Conference on BioInformatics and BioEngineering (IEEE BIBE 2008) 2008

[B41] Carneiro S Rocha I Ferreira EC Application of a genome-scale metabolic model to the inference of nutritional requirements and metabolic bottlenecks during recombinant protein production in Escherichia coli Microb Cell Fact 2006 5 Suppl 1

[B42] Carneiro S Amaral AL Veloso AC Dias T Peres AM Ferreira EC Rocha I Assessment of physiological conditions in E. coli fermentations by epifluorescent microscopy and image analysis Biotechnol Prog 2009 25 882 891 10.1002/btpr.134 19496165

[B43] Carneiro S Villas-Bôas S Rocha I Ferreira EC Applying a metabolic footprinting approach to characterize the impact of the recombinant protein production in Escherichia coli Advances in Soft Computing edition 2010 193 200

[B44] Lourenço A Carreira R Glez-Peña D Méndez JR Carneiro S Rocha LM Díaz F Ferreira EC Rocha I Fdez-Riverola F Rocha M BioDR: Semantic indexing networks for biomedical document retrieval Expert Systems with Applications 2010 37 3444 3453 10.1016/j.eswa.2009.10.044

[B45] Keseler IM Collado-Vides J Santos-Zavaleta A Peralta-Gil M Gama-Castro S Muniz-Rascado L Bonavides-Martinez C Paley S Krummenacker M Altman T Kaipa P Spaulding A Pacheco J Latendresse M Fulcher C Sarker M Shearer AG Mackie A Paulsen I Gunsalus RP Karp PD EcoCyc: a comprehensive database of Escherichia coli biology Nucleic Acids Res 2010 10.1093/nar/gkq1143 PMC3013716 21097882

[B46] Chatr-aryamontri A Kerrien S Khadake J Orchard S Ceol A Licata L Castagnoli L Costa S Derow C Huntley R Aranda B Leroy C Thorneycroft D Apweiler R Cesareni G Hermjakob H MINT and IntAct contribute to the Second BioCreative challenge: serving the text-mining community with high quality molecular interaction data Genome Biol 2008 9 Suppl 2 S5 10.1186/gb-2008-9-s2-s5 18834496 PMC2559989

[B47] Lourenço A Carreira R Carneiro S Maia P Glez-Peña D Fdez-Riverola F Ferreira EC Rocha I Rocha M @Note: A workbench for Biomedical Text Mining Journal of Biomedical Informatics 2009 10.1016/j.jbi.2009.04.002 19393341

[B48] Brants T Inter-annotator agreement for a German newspaper corpus In the Second International Conference on Language Resources and Evaluation (LREC-2000) 2000 1435 1439

